# Effectiveness of *Moringa oleifera*, chitosan, and alum as adsorbents in lake water treatment

**DOI:** 10.1007/s11356-026-37492-7

**Published:** 2026-02-10

**Authors:** Yvan Anderson Tchangoue Ngandjui, Paul Atabong Agendia, Alex Tawanda Kuvarega, Volodymyr Tarabara, Titus Alfred Makudali Msagati

**Affiliations:** 1 College of Science, Engineering and Technology, Institute for Nanotechnology and Water Sustainability, University of South Africa, Johannesburg, South Africa; 2 Faculty of Sciences, University of Yaounde I, Yaounde, Cameroon; 3 Department of Civil and Environmental Engineering, Michigan State University, East Lansing, USA

**Keywords:** Coagulation, Adsorption, *Moringa oleifera*, Chitosan, Alum, Lake water, Water treatment

## Abstract

This study evaluated the effects of *Moringa oleifera* (MO), chitosan, and alum as adsorbents on the physicochemical properties of water collected from Lake Florida in Johannesburg, South Africa. The lake water was subjected to three different treatments using jar tests at concentration dosages of 25, 30, and 35 mL and settling times of 30, 60, and 90 min. The water treated with adsorbents significantly reduced turbidity (*p* < 0.05) with removal efficiencies of 99.33% for MO (30 mL, 30 min), 99.22% for chitosan (35 mL, 60 min), and 99.60% for alum (25 mL, 60 min). Dissolved oxygen increased significantly (p < 0.05) from 2.06 ± 0.02 mg/L to 3.24 ± 0.01 mg/L with chitosan (25 mL, 90 min) and MO (35 mL, 90 min), and to 3.15 ± 0.01 mg/L with alum (25 mL, 60 min). Sulfate levels increased with MO from 65.00 ± 1.00 mg/L to 200.67 ± 0.58 mg/L (35 mL, 90 min)**,** while alum caused an initial decrease to 49.67 ± 0.58 mg/L (25 mL, 30 min), followed by an increase to 71.33 ± 0.58 mg/L. Furthermore, total dissolved solids and conductivity increased with MO, whereas chitosan and alum caused no significant changes. However, a slight pH reduction was noted, with no significant nitrate alteration. Based on principal component analysis, the key factors driving water quality variations in the dataset were treatment type and retention time, with parameters such as pH, conductivity, and sulfate being strong indicators of treatment efficiency. Dissolved oxygen and nitrate were more dependent on treatment time. These findings provide insights into the performance of different adsorbents and their impacts on lake water quality.

**Graphical Abstract:**

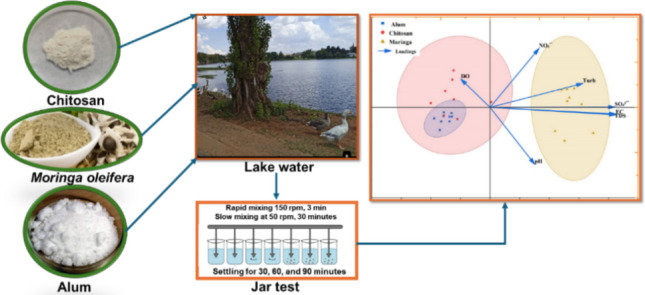

**Supplementary Information:**

The online version contains supplementary material available at 10.1007/s11356-026-37492-7.

## Introduction

Access to safe and clean water remains a significant problem for many countries worldwide, particularly in rural areas where conventional water treatment facilities may be limited (Werku & Woldeamanuel 2025). The quest for effective and sustainable water treatment methods has led to increased interest in natural coagulants as alternatives to traditional chemical agents. Although conventional coagulants, such as aluminum sulfate (alum), are frequently used as synthetic coagulants to purify water because of their rapid aggregation and formation of heavy flocs, well-documented concerns about their costs, generation of voluminous toxic sludge, residual aluminum in treated water, and tendency to depress water pH have catalyzed the search for sustainable and natural alternatives (Ngandjui et al. 2025; Mitiku 2020). In contrast, natural coagulants are generally biodegradable, relatively inexpensive, low in toxicity, and produce significantly less sludge, aligning with principles of green chemistry and circular economy (Kakkar et al. 2025; Cardoso Valverde et al. 2018).

Among the plethora of natural materials investigated, chitosan and *Moringa oleifera* seeds have emerged as two of the most promising candidates, yet they represent fundamentally different classes of biopolymers with distinct mechanisms and application challenges. *Moringa oleifera* seed (MOs) is obtained from a drought-resistant “miracle tree.” Its effectiveness is primarily attributed to water-soluble cationic proteins that act as natural polyelectrolytes, enabling charge neutralization and bridging of colloidal particles (Bancessi et al. 2022; Dandesa et al. 2023). Extensive laboratory studies have confirmed its efficacy in reducing turbidity and pathogens in synthetic and wastewater streams, often reporting the advantage of not altering the pH of the treated water (Al-Manhel et al. 2018; Rasheed et al. 2023). However, a critical literature gap persists regarding its performance consistency and optimal dosing in complex, natural surface waters with variable ionic strength and natural organic matter content, which can interfere with protein activity (Nhut et al. 2021).

Chitosan, a derivative of chitin, is a natural polycationic linear polysaccharide. Its mechanism extends beyond simple coagulation; the protonable amine groups enable not only charge neutralization but also chelation of specific anionic pollutants like phosphates and heavy metals (Bhatt et al. 2023). It is biocompatible, biodegradable, and has adaptable surface chemistry. However, a key operational limitation noted in the literature is its solubility dependency on pH; it requires acidic conditions for dissolution, which may necessitate pH adjustment before use in neutral lake water, adding a step to the treatment process (Piekarska et al. 2023). Furthermore, while its adsorption capacity for specific ions is well documented in controlled single-solute systems, its integrated performance in multi-contaminant lake water matrices against a standard like alum is less explored (Gonçalves et al. 2024).

Direct comparative studies between these natural options and alum, while insightful, often focus on a single parameter (e.g., turbidity removal) under idealized conditions. For instance, studies show alum’s high efficiency but confirm its pH-lowering effect and sludge volume, whereas *Moringa oleifera* preserves pH but may require higher doses (Salazar Gámez et al. 2015; Ngandjui Tchangoue et al. 2019). A comprehensive, side-by-side evaluation under identical, realistic conditions assessing a broader suite of critical water quality parameters (e.g., turbidity, dissolved oxygen, nutrients, ionic content) and practical factors like optimal dose, settling time, and resultant sludge volume is lacking. This gap is particularly acute in regional contexts like South Africa, where local water chemistry can profoundly influence coagulant performance.

Therefore, although several studies have reported the use of natural coagulants for wastewater treatment, insufficient attention has been paid to surface water in South Africa. Further research focusing on the effectiveness of *Moringa oleifera*, chitosan, and alum as adsorbents specifically in the context of lake water treatment is warranted to develop optimized, eco-friendly water purification strategies. This study aims to fill this gap by providing a holistic comparative analysis. It uses *Moringa oleifera* seeds, chitosan, and alum to assess the removal efficiency of turbidity, dissolved oxygen (DO), total dissolved solids (TDS), conductivity, nitrate, and sulfate under the influencing parameters of coagulant dosage and settling time in lake water. Furthermore, principal component analysis (PCA) is applied in coagulation studies to identify key influencing parameters and optimize processes, typically for conventional coagulants in wastewater (Ou et al. 2014). This study’s novel application uses PCA as a diagnostic tool to decode and compare the distinct mechanistic signatures of alum, chitosan, and *Moringa oleifera* in lake water. It moves beyond reporting removal efficiencies to reveal how each coagulant uniquely alters the multivariate water chemistry profile. This systems-level analysis provides insight into their fundamental modes of action. Consequently, the findings offer a refined trade-off analysis to guide coagulant selection for sustainable lake water remediation.

## Materials and methods

### Surface water sampling

The surface water samples were collected in the middle of November 2024 from Florida Lake water located in Johannesburg, South Africa (Coordinates 26°10′42.2″S–27°54′23.8″E). This water was collected at a depth of 10–30 cm from the top surface of the water body using clean 1 L glass bottles with Teflon-lined caps and transported to the laboratory in a cooler bag.

### Reagents and feed water

*Moringa oleifera* seeds were obtained from retailers at Mokolo market (Yaounde, Cameroon). Reagent grade NaCl, chitosan (low molecular weight, Deacetylated chitin, Poly(D-glucosamine)), and alum (Al_2_(SO_4_)_3_.18H_2_O) were procured from Merck, South Africa. All aqueous solutions were prepared using ultra-pure water (resistivity of ~ 18.2 mΩ.cm and pH ~ 6.998) from a MilliQ water purification system (Molsheim, France).

### Preparation of *Moringa oleifera*-derived coagulant, chitosan coagulant, and alum solution

The seeds of *Moringa oleifera* were de-shelled and dried at ambient temperature before milling. The white kernels were powdered using a milling machine (POWTEQ-BM6Pro, rotation 150 rpm and time 20 min). Milled seed fragments were separated into fine powder using a sieve of 600 µm size range, which is within the range recommended to achieve better coagulation efficiency (Landázuri et al. 2018). The powder was then stored in a sterile capped bottle.

The preparation of stock solutions was designed to achieve effective concentration ranges reported in the literature while considering material solubility and practical handling. The coagulant extract of *Moringa oleifera* was made by adding 2.5 g of the powdered MO seeds to 250 mL of an aqueous solution containing 10 mM NaCl and stirring using a magnetic stirrer for 15 min. This produces a 10 g/L stock extract, a concentration commonly used to facilitate dose–response studies in jar tests (Ghebremichael et al. 2005). The mixture was then filtered using a sterile syringe filter with a pore size of 0.45 μm to remove seed fragments (Ghebremichael et al. 2005; Murali et al. 2022).

Two additional coagulants, chitosan and alum, were also evaluated by preparing solutions with various dosages and under the same conditions for comparison. The chitosan solution was prepared by dissolving 0.8 g of chitosan powder in 5 mL of 1% acetic acid. This mass was selected to create an 8 g/L chitosan stock solution in 1% acetic acid, a concentration that ensures complete dissolution and is within the effective range for coagulation studies (El-Hefian et al. 2010). The mixture was stirred using a magnetic stirrer for 15 min, topped up to 100 mL with DI water, and then stirred again for two hours. The obtained solution was then filtered to remove suspended particles. Air bubbles were eliminated by keeping the solution at room temperature for 2 h (El-Hefian et al. 2010; Abraham et al. 2016; Zuhannisa et al. 2017). Aluminum sulfate (alum) [Al_2_(SO_4_)_3_.18H_2_O] was used in this study as the inorganic coagulant, and it was prepared by dissolving 0.4 g in 1 L of ultra-pure water. This results in a 0.4 g/L (400 mg/L) alum stock solution, representing a typical mid-range concentration for direct comparative jar testing with natural coagulants. The solution was stirred for five minutes using a magnetic stirrer for complete dissolution (Murali et al. 2022).

For the jar tests, the applied coagulant dosage volumes of 25 mL, 30 mL, and 35 mL per litre of lake water were selected based on preliminary range-finding experiments. These ranges encompass the typical optimal doses reported for each coagulant type in surface water treatment, allowing for a direct comparison of their performance and the identification of their specific optimum within this study’s context.

### Experimental jar test procedure

The standard three-step process of coagulation, tapered flocculation, and sedimentation was followed while using the programmable jar test apparatus (CAT REF W1-A, Serial number 38356-001, VELP Scientifica) and six 2L jars (beakers). The study employed a full factorial experimental design to enable statistical analysis, with the independent factors being: (1) Coagulant type (*Moringa oleifera* seed powder, Chitosan, Alum), (2) Coagulant dosage, and (3) Settling time**.** The concentrations (dosages), different times, and rotational speeds of the stirring paddle were determined as optimal based on the literature (Ngandjui Tchangoue et al. 2019; Desta and Bote 2021; Murali et al. 2022; Silva 2023) and on preliminary tests. After settling, the treated lake water samples were collected in each jar for analysis. Each unique treatment combination was conducted in three independent, randomized replicate jar tests (*n* = 3). The tests were carried out at room temperature following the procedure below:

- Mixing the lake water in beakers at 150 rpm for 3 min (rapid speed) while adding coagulant.
- Reducing the mixing to 50 rpm and continuing the slow mix for 30 min while adding the flocculant.
- Turning off the mixer and allowing settling to occur for 30 min, 60 min, and 90 min.
- After settling, extracting the supernatant, and keeping the water for further analysis.

These parameters were kept the same for jar tests with *Moringa oleifera–*based coagulants, chitosan, and alum: the coagulant dosage volumes used were the only significant difference between these tests.

### Water analysis and statistical analysis

Fresh lake water and coagulated samples were analyzed for their pH, DO (Dissolved Oxygen), conductivity, and TDS (Total Dissolved Solids) using the multiparameter HANNA HI9829 (Woonsocket, Rhode Island, USA). On the other hand, the turbidity was determined using a Turbidity Meter TB400 (EXTECH Instruments, Taiwan). Nitrate (NO_3_^−^) and sulfate (SO_4_^2−^) were measured using a Spectroquant Pharo 300 M (Merck KGaA, Darmstadt, Germany). The monitoring of organic content was done by measuring UV absorption using a UV spectrometer (Shimadzu, UV-1800, Japan) at a wavelength between 200 and 450 nm.

The structured dataset generated from the full factorial experimental design was subjected to statistical analysis. The effect of various parameters on coagulation performance was analyzed using a multiparametric ANOVA test to assess statistical significance, with the significance level set at *α* = 0.05. Pairwise comparisons between treatments were conducted using the least significant difference (LSD) test to determine significant differences (the letters in the boxplot represent the results of statistical comparisons used to determine significant differences between the means of the different treatments). Furthermore, principal component analysis (PCA) was applied to reduce dimensionality, extract key trends, and highlight correlations among the effects of natural coagulants on water quality parameters, revealing distinct groupings and interdependencies. All statistical analyses and data visualizations were performed using the OriginPro 2024 software (OriginPro Corporation, New York, NY, USA).

## Results and discussion

### Effects of treatments on the turbidity

**Fig. 1:**
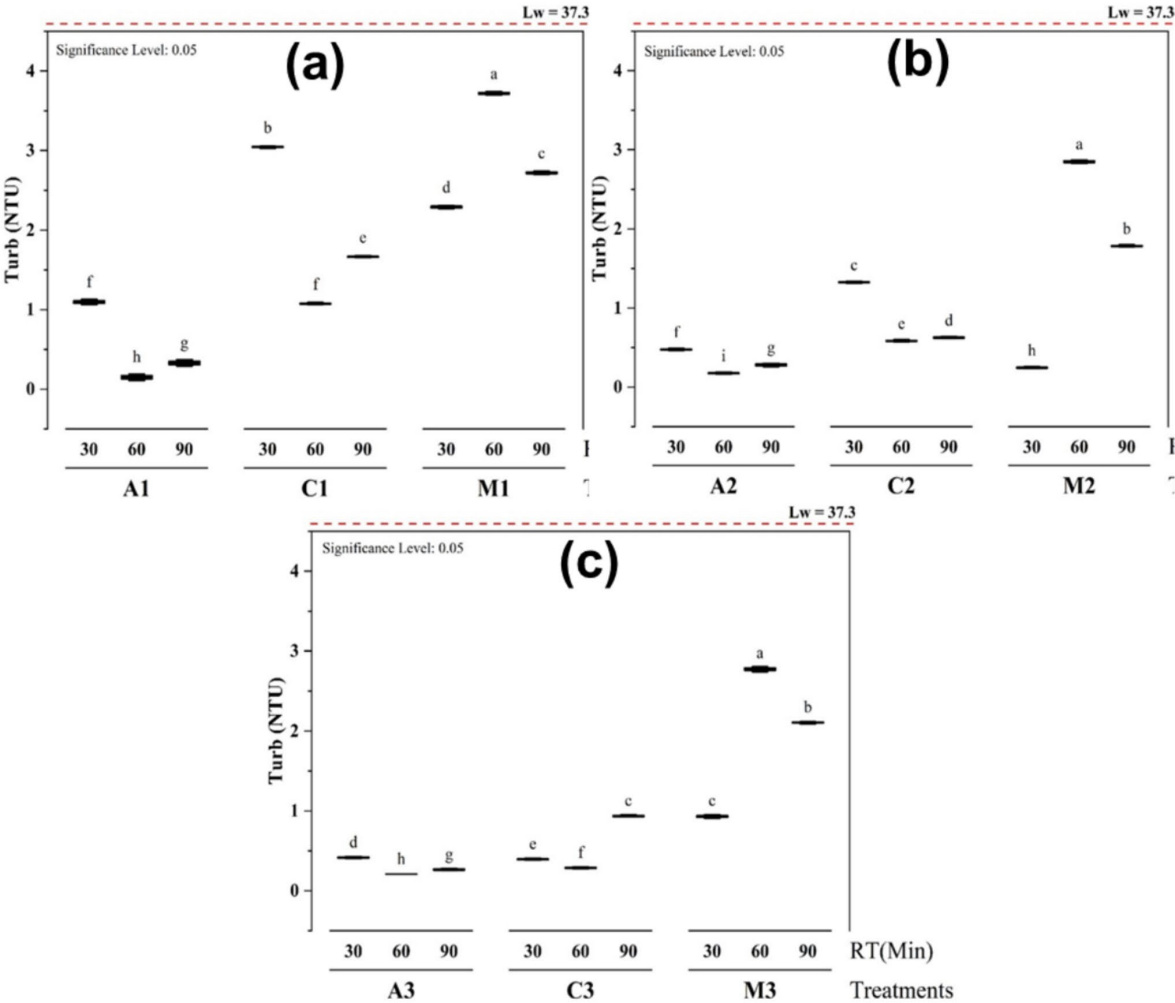
Effects of different treatments (**a** 25 mL, **b** 30 mL, and **c** 35 mL) on the turbidity at different coagulant dosage volumes and time of decantation

The effects of the different treatments on turbidity were compared with the initial turbidity of the fresh lake water (Lw), which was 37.33 ± 1.08 NTU. The treatments tested were alum (A), chitosan (C), and *Moringa oleifera* (M) at various coagulant dosage volumes of 25 mL (1), 30 mL (2), and 35 mL (3), and applied at 30, 60, and 90 min (Fig. 1).

There is a significant difference at p ≤ 0.05 with different levels of significance (letters from a to i) between the adsorbents and the time of decantation. However, there is no significant difference between the treatment with alum after 30 min and chitosan after 60 min, while using 25 mL (Fig. 1a), and the treatment with chitosan after 90 min and *M. oleifera* after 30 min, while using 35 mL (Fig. 1c) because they are sharing the same letters (f and c respectively).

At the volume of 25 mL (Fig. 1a), alum (A1) treated water had the lowest turbidity (0.15 ± 0.02 NTU), followed by chitosan (C1) treated water (1.08 ± 0.01 NTU) after 60 min. *Moringa oleifera* (M1) treated water exhibited the highest turbidity (2.29 ± 0.01 NTU) after 30 min, suggesting that it was less effective in reducing turbidity compared to alum and chitosan. The turbidity increases with *Moringa oleifera* and chitosan may be due to fine particle release, supra-optimal dose restabilization, and formation of slow-settling micro-flocs (Vigneshwaran et al. 2020). In contrast, alum forms dense, fast-settling hydroxide flocs via sweep coagulation, yielding lower residual turbidity (Dayarathne et al. 2022).

With a volume of 30 mL (Fig. 1b), the turbidity of alum (A2) treated water decreased to 0.18 ± 0.01 NTU after 60 min, showing the most significant reduction resulting in the clearest water. On the other hand, *M. oleifera* (M2) treated water exhibited its most important performance after 30 min with a turbidity of 0.25 ± 0.01 NTU. Chitosan (C2) treated water followed with turbidity at 0.59 ± 0.01 NTU after 60 min.

At the volume of 35 mL (Fig. 1c), the turbidity of alum (A3) treated water was still the best with a slight decrease to 0.21 ± 0.01 NTU, followed by chitosan (C3), which showed its best performance with a significant decrease to 0.29 ± 0.01 NTU after 60 min. In contrast, *M. oleifera*-treated water exhibited a turbidity of 0.93 ± 0.01 NTU after 30 min, indicating a further decrease in its effectiveness at clearing the water. The performance decline of *M. oleifera* at the highest dosage (35 mL) further supports the restabilization hypothesis, where an overdose provides excessive positive charge, preventing effective particle aggregation and leading to higher residual turbidity.

In conclusion, it was observed that alum, chitosan, and *Moringa oleifera* reduced the turbidity of the initial lake water. These adsorbents can destabilize and aggregate suspended particles, which causes them to settle out of the water and reduce its turbidity. The lowest turbidity values were 0.15 NTU for alum (A1) after 60 min, 0.25 NTU for *M. oleifera* (M2) after 30 min, and 0.29 NTU for chitosan (C3) after 60 min. They are below the 5 NTU limit recommended by the World Health Organization for drinking water, and the reduction is greater than 99%. Alum treatment proved to be the most effective in reducing turbidity, consistently maintaining low turbidity levels across all time points. However, the natural coagulant *Moringa oleifera* showed initial effectiveness but became less efficient as time progressed, with turbidity increasing significantly by 60 and 90 min. This temporal increase for *M. oleifera* could be due to the breakup of initially formed aggregates or the continued release of fine organic colloids from the seed matrix over the extended settling period (Precious et al. 2021). Chitosan had a moderate effect, initially reducing turbidity but failing to achieve as low a level as alum by the end of the experiment. These findings suggest that alum is the most effective treatment for reducing turbidity, while *M. oleifera* can be used at a certain concentration and with a specific settling time to reach a good percentage of turbidity reduction. Crucially, the results highlight that the optimal dose window for natural coagulants like *Moringa oleifera* is narrower than for alum; exceeding it can paradoxically increase turbidity due to colloidal restabilization or the introduction of new particulates.

These results are like those of Murali et al. (Murali et al. 2022) and Silva and Oliveira (Silva and Oliveira 2024), where *Moringa oleifera* best dosage was also 30 mL. Moreover, they are in line with other researchers who have figured out that MO can remove 90%–100% turbidity from wastewater (Eman et al. 2014; Salazar Gámez et al. 2015; Gutierrez Herrera et al. 2024).

### Effects of treatments on the residual organics

**Fig. 2:**
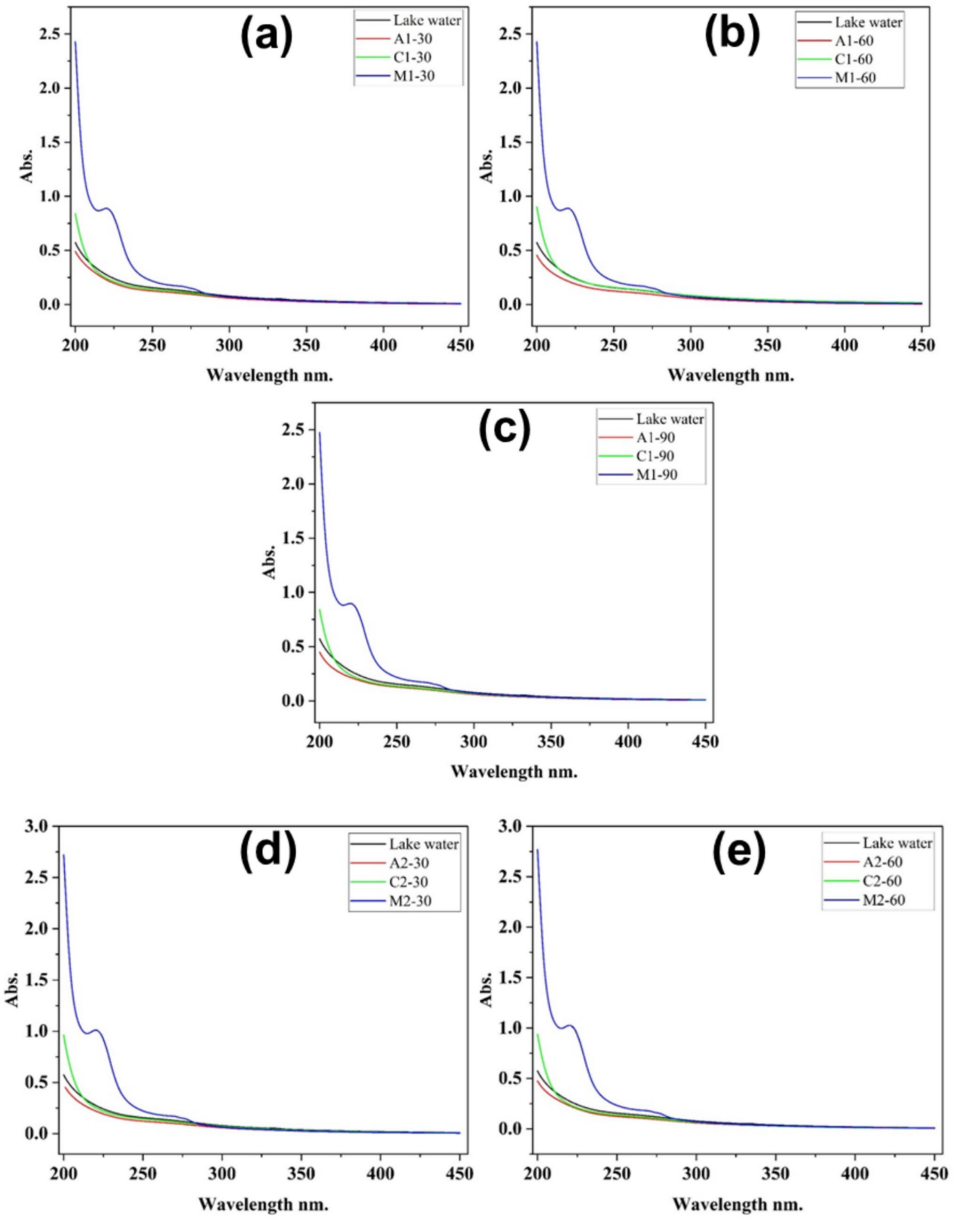

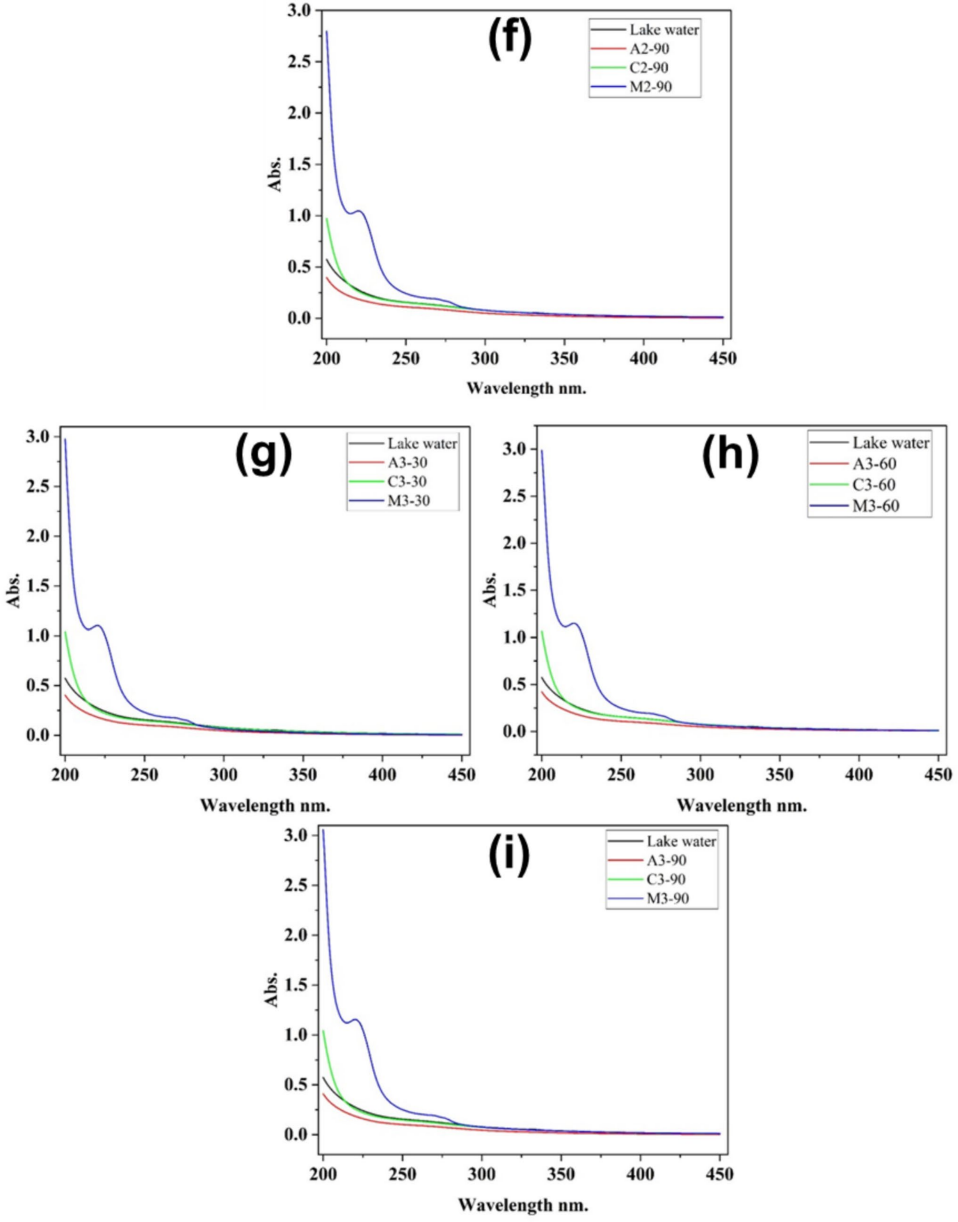
Effects of different treatments at the dosage volume of 25 mL (**a**, **b**, **c**), 30 mL (**d**, **e**, **f**), and 35 mL (**g**, **h**, **i**) on organics

The lake water was treated with different coagulants (alum, chitosan, and *Moringa oleifera*) at different dosages, 25 mL (1), 30 mL (2), and 35 mL (3), and over varying reaction times (30, 60, and 90 min). Figure 2 shows that the treatment time and the type of coagulant affect the water treatment efficiency.

In Fig. 2, we observed a progressive increase in absorbance and the appearance of a specific wavelength at 221 nm while treating lake water with *Moringa oleifera* from 2.43 to 2.48 absorbance units (a, b, c), from 2.72 to 2.80 (d, e, f), and from 2.98 to 3.06 (g, h, i). The increase and the specific peak could be attributed to the leaching of soluble organic components, such as proteins or other biomolecules, from the Moringa seed matrix into the water during the treatment process (Camacho et al. 2017; Chales et al. 2022). While *Moringa oleifera* is highly effective for particulate destabilization, this result reveals a potential trade-off: it may contribute to an elevated concentration of dissolved organic matter, which is a parameter of concern for downstream disinfection processes.

In general, Fig. 2 shows an increase in absorbance with increasing the dosage of chitosan and MO, and a slight decrease with alum. These observations can be justified by the fact that chitosan and *Moringa oleifera* are biodegradable polymers that are closely related to organics (Al-Manhel et al. 2018; Al-Jadabi et al. 2023). In Fig. 2, we observed that *M. oleifera* (blue line) had the maximum absorbance among the coagulants, indicating that UV-absorbing molecules are not entirely removed, while the lower absorbance values of alum (red) and chitosan (green) indicate their effectiveness in reducing dissolved organic matter and other pollutants. These findings are similar to other studies evaluating coagulants in water treatment, where it was shown that *Moringa oleifera* may be less efficient than alum and chitosan in removing dissolved organic carbon and UV-absorbing compounds (Ndabigengesere et al. 1995; Sánchez-Martín et al. 2010). It was also observed that across all results, absorbance decreases progressively with time (from 30 to 90 min), supporting the idea that longer reaction times enhance coagulation and pollutant removal efficiency. After 90 min, the differences become more pronounced, with alum and chitosan achieving the best UV absorption reductions.

### Influence of pH using different treatments

The effects of different treatments involving alum (A), chitosan (C), and *Moringa oleifera* (M) on the pH of lake water (Lw) were assessed over different periods and dosage volumes (from 30 to 90 min, and from 25 to 35 mL), with the control water (lake water) having an initial pH of 7.12 ± 0.01.

**Fig. 3:**
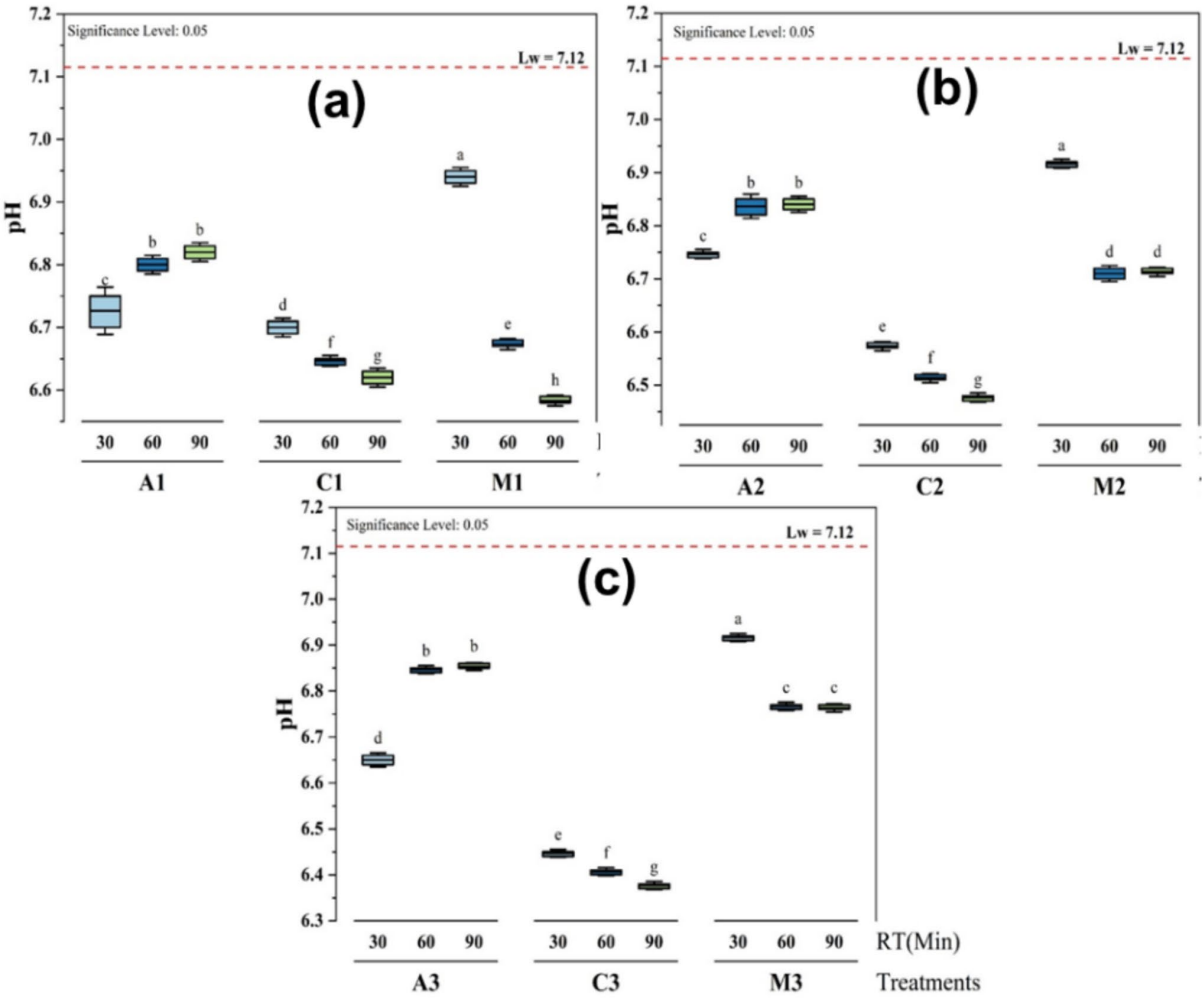
Effects of different treatments (**a** 25 mL, **b** 30 mL, and **c** 35 mL) on the pH at different coagulant dosage volumes and time of decantation

It was observed that the pH values were less than the initial one, and that there is a significant difference at p ≤ 0.05 with different levels of significance (letters from a to h) between the adsorbents and the time of decantation (Fig. 3). However, there is no significant difference between the treatment with alum after 60 and 90 min for the three different volumes (Fig. 3a, b, and c) and with *M. oleifera* after the same time, but only with volume 30 mL (Fig. 3b) and 35 mL (Fig. 3c).

The treatment with *M. oleifera* at the dosage volume of 25 mL showed the closest value to the initial one, with a pH of 6.94 ± 0.01 (Fig. 3a), which was higher than the other treatments. At 90 min, chitosan at the dosage volume of 35 mL showed the lowest pH with a value of 6.38 ± 0.01 (Fig. 3c).

Overall, the results indicate that *M. oleifera* maintained the highest pH among all treatments, although it decreased over time. The values were within the range of 6.5–9.5 as recommended by the World Health Organization for drinking water while treating with alum and *Moringa oleifera* at different volumes (Fig. 3), but below the range after the treatment with chitosan at the volume of 35 mL (Fig. 3c). Alum had a more stable pH, with minimal fluctuation, while chitosan caused the greatest decrease in pH, particularly over time. The data suggest that *M. oleifera* was the most effective treatment for maintaining a higher pH, while chitosan had the most pronounced acidifying effect. Alum was able to maintain moderate pH values throughout the experiment, making it suitable for applications where pH stability is necessary.

These data corroborate the findings of various authors who report that *M. oleifera* does not have a significant effect on pH variation after a short time, while the pH of water decreases with increasing chitosan concentration (Al-Manhel et al. 2018; Gutierrez Herrera et al. 2024).

### Influence of DO using different treatments

**Fig. 4:**
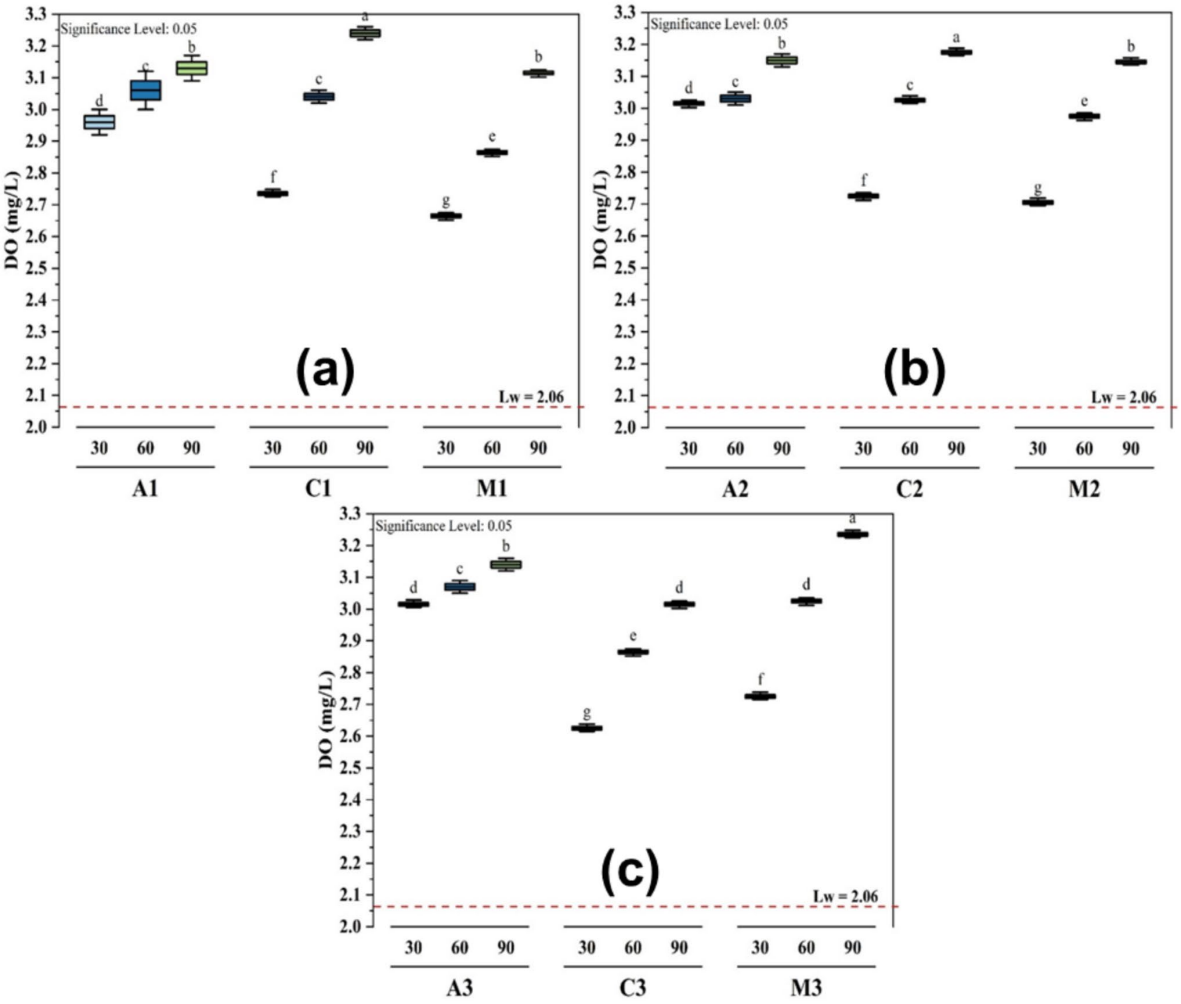
Effects of different treatments (**a** 25 mL, **b** 30 mL, and **c** 35 mL) on the DO at different coagulant dosage volumes and time of decantation

During all the treatments, the dissolved oxygen (DO) levels in the lake water (Lw) increased (Fig. 4) from 2.06 ± 0.02 mg/L to a maximum of 3.24 ± 0.01 mg/L while treated with chitosan at a dosage volume of 25 mL (Fig. 4a) and a volume of 35 mL with *M. oleifera* (Fig. 4c), both after 90 min. DO serves to evaluate whether the coagulants impart a significant oxygen demand or adversely affect this key parameter for aquatic life, thereby providing insight into the environmental compatibility of the treatment (Shan et al. 2017)**.** The lowest DO level of *M. oleifera* treatment is shown in Fig. 4a with a value of 2.66 ± 0.01 mg/L after 30 min, and the chitosan in Fig. 4c with a value of 2.63 ± 0.01 mg/L after 30 min. The dissolved oxygen levels were lower than the minimum value of 5 mg/L required by the World Health Organization for drinking water. A significant difference at p ≤ 0.05 with different levels of significance (letters from a to g) between the adsorbents and the time of decantation was observed, but no significance between the treatment with alum and *M. oleifera* after 90 min at dosages of 25 mL and 30 mL, and between alum and chitosan after 60 min at the same dosages (Fig. 4a and b).

Overall, the *M. oleifera* and chitosan treatments exhibited the most consistent improvement in DO, particularly at the later stages of the experiment. The *M. oleifera* treatment showed gradual improvement over time, but remained lower than alum and chitosan. The alum treatment showed a moderate but steady increase in DO, suggesting a mild positive effect.

These findings suggest that while all treatments had some effect on DO levels, alum was the most effective in increasing oxygen concentration in the water in the beginning, but *M. oleifera* and chitosan took over that performance after 90 min. These results show that the increase was proportional to the dosage used and the settling time (Eman et al. 2014).

### Influence of conductivity using different treatments

**Fig. 5:**
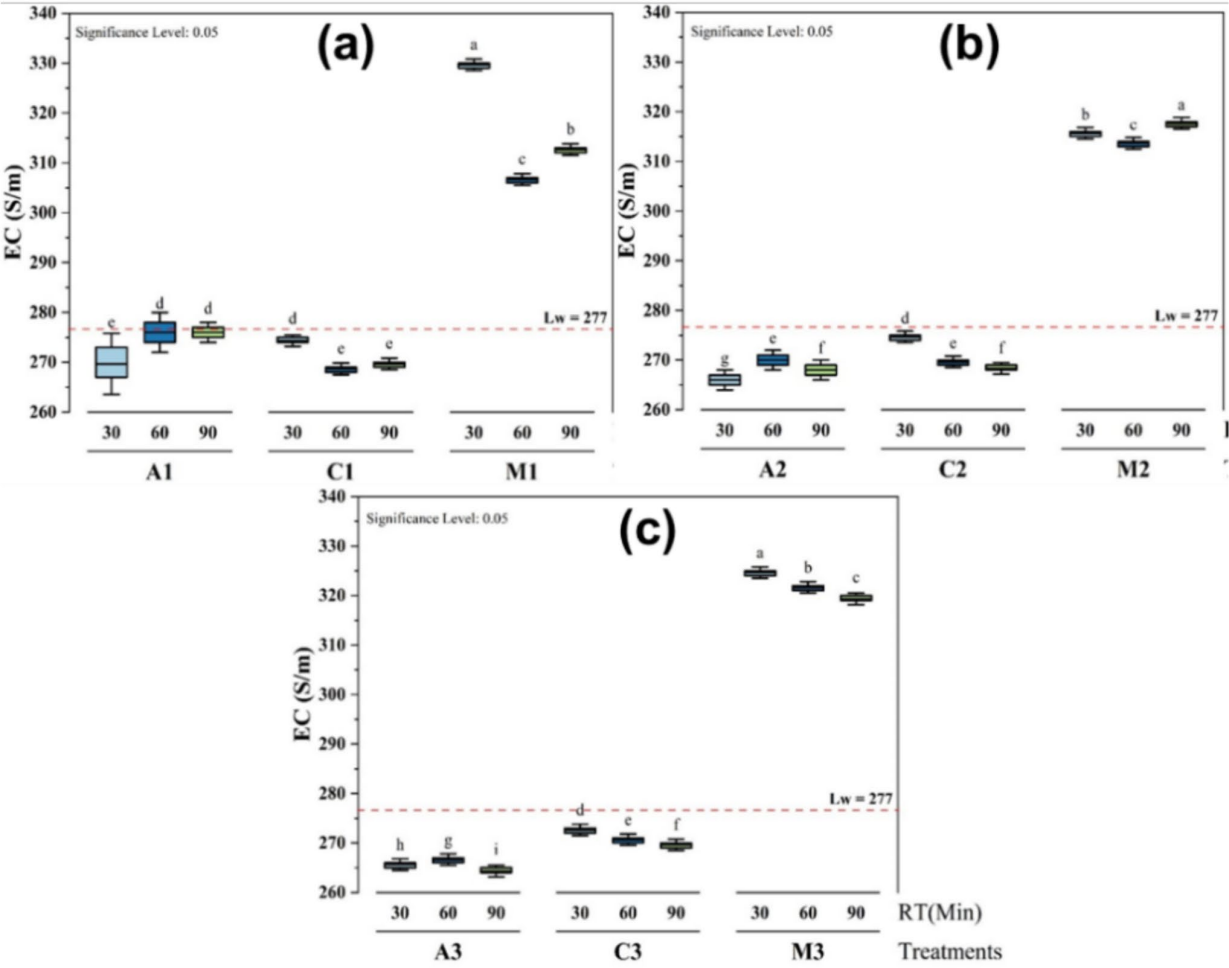
Effects of different treatments (**a** 25 mL, **b** 30 mL, and **c** 35 mL) on the conductivity at different coagulant dosage volumes and time of decantation

The baseline conductivity of the lake water (Lw) was 277 µS/m, providing a reference for comparing the effects of the different treatments over time (30, 60, and 90 min) at variable dosage volumes and with different levels (letters from a to i) of significance (Fig. 5). At 30 min and the dosage volume of 25 mL, the *M. oleifera* treatment (Fig. 5a) had the highest conductivity at 329.67 ± 0.58 µS/cm, which was significantly higher than all other treatments (p < 0.05). The lowest conductivity under the baseline was observed when using alum at the dosage volume of 35 mL (Fig. 5c), with a value of 264.33 ± 0.58 µS/cm at 90 min. All the obtained values were below 1000 *µ*S/cm, which is the recommended limit of the World Health Organization for drinking water.

Overall, the results indicate that *M. oleifera* had the most significant impact on increasing EC, with values consistently higher than the control and the other treatments at all time intervals. Chitosan also caused a moderate increase in EC, but its effect was less pronounced than *M. oleifera*. In contrast, alum had the least effect on EC, maintaining values below and closer to the control. This suggests that *M. oleifera* is the most effective treatment for increasing EC, while alum has the least impact on conductivity, with values consistently lower than the control.

These findings can be explained by the minerals found in *M. oleifera* seeds, which dissolve in water and increase water conductivity. Moreover, the saline extraction (NaCl) of the seeds of *M. oleifera* may carry an electrical charge and result in the dispersion of some mineral ions and inorganic compounds in water, which might enhance the water ionic conductivity by reaction between charged metals (Shan et al. 2017; Varsani et al. 2022).

These results are similar to Al-Manhel et al. (2018) who showed that the electrical conductivity of water decreased with increasing chitosan concentrations. In the same line, this observation is in agreement with the findings of Shan et al. (2017) who reported an increase in conductivity of wastewater with increasing concentration of *M. oleifera* seeds, which may be attributed to the increase in cationic polyelectrolyte in *M. oleifera* seeds. However, the current findings contradict (Vunain et al. 2019), which showed that conductivity values decreased as *M. oleifera* coagulant concentrations increased.

### Influence of TDS using different treatments

**Fig. 6:**
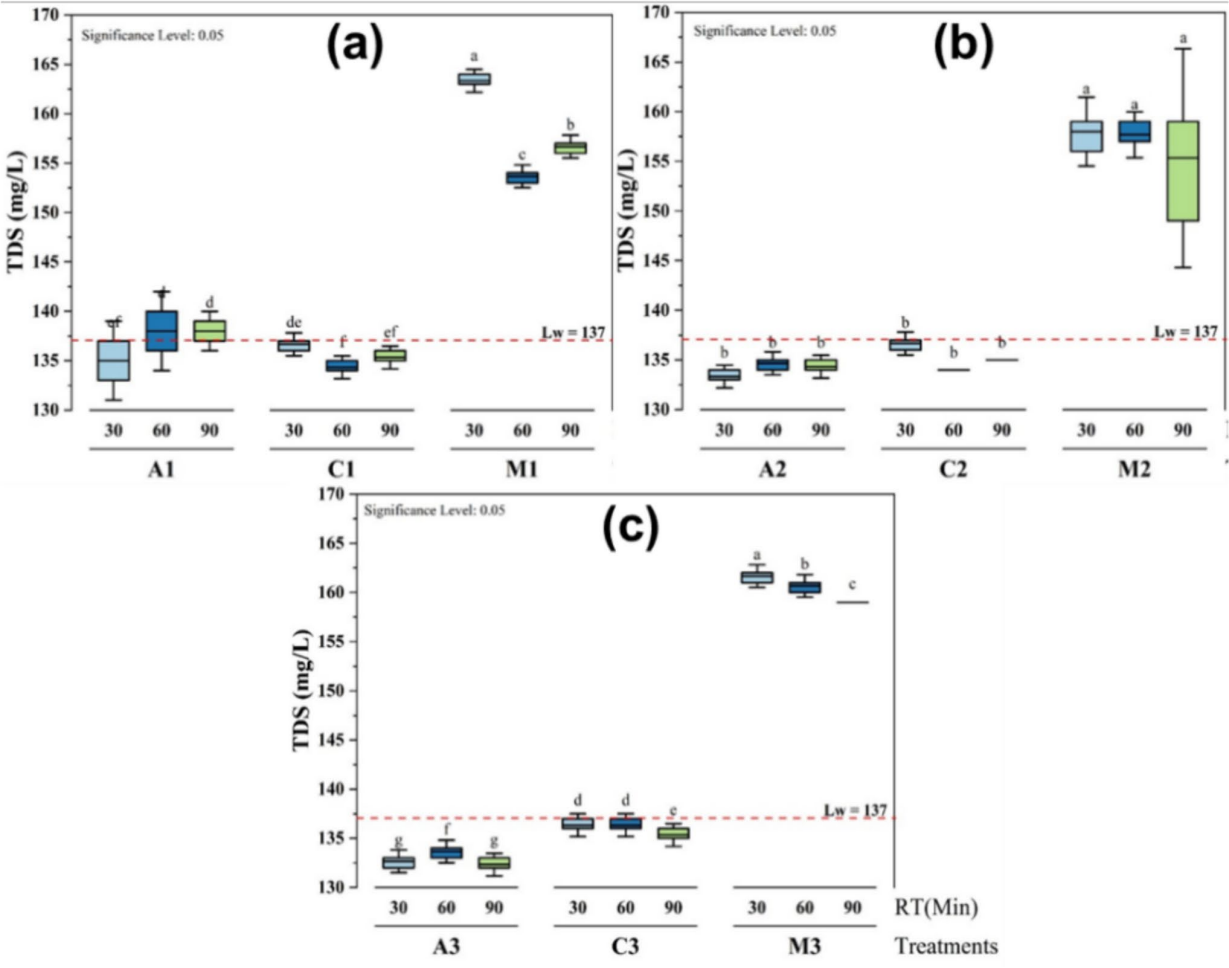
Effects of different treatments (**a** 25 mL, **b** 30 mL, and **c** 35 mL) on the TDS at different coagulant dosage volumes and time of decantation

The effects of the different treatments on TDS over time (30, 60, and 90 min) and at different dosage volumes gave some significant differences (letters from a to g) compared to the TDS value 137.00 ± 2.00 mg/L of the lake water (Fig. 6). It was observed that there is only a significant difference at p ≤ 0.05 during the treatment with *M. oleifera* over time and at volumes of 25 mL (Fig. 6a) and 35 mL (Fig. 6c).

The results showed that *M. oleifera* significantly increased TDS levels throughout the experiment to 163.33 ± 0.58 mg/L after 30 min (Fig. 6a), likely due to the release of dissolved organic and inorganic compounds. Alum treatment had a minimal effect with a value of 132.33 ± 0.58 mg/L, keeping TDS close to the control value, with slight fluctuations over time (Fig. 6c).

Overall, the results suggest that *M. oleifera* treatment consistently led to the highest TDS values across all time points, indicating that it contributed the most to the increase in TDS. In contrast, the alum and chitosan treatments showed minimal changes in TDS, with values remaining relatively stable and not significantly differing from each other. These findings indicate that *M. oleifera* is the most effective treatment for increasing TDS, while alum and chitosan have less impact on the TDS levels in the water. The values of TDS before and after treatment were below the limit of 1000 mg/L recommended by the World Health Organization for drinking water.

However, these results were not in agreement with the findings of some authors who stated that total dissolved solids were reduced after the treatment with *M. oleifera* seeds (Varsani et al. 2022; Kenea et al. 2023). This may have been due to the pre-treatment step used in the experiment during the preparation of the *M. oleifera* solution with NaCl. This is made possible by the ions from the dissolved particles in the water samples, which give the water its electrical conductivity. Additionally, salt is one of the components of TDS, and conductivity is proportional to salinity. As a result, the levels of conductivity and TDS were increased after the treatment (Shan et al. 2017). This observation is consistent with the results of Shan et al. (2017), who found that the TDS content of water increased as the concentration of *M. oleifera* seed increased. According to Kitheka et al. (2022), there is a strong positive correlation between the concentration of *M. oleifera* seed coagulant and TDS, and the TDS of water significantly increases as the concentration of *M. oleifera* seed powder increases.

### Influence of sulfate and nitrate using different treatments

**Fig. 7:**
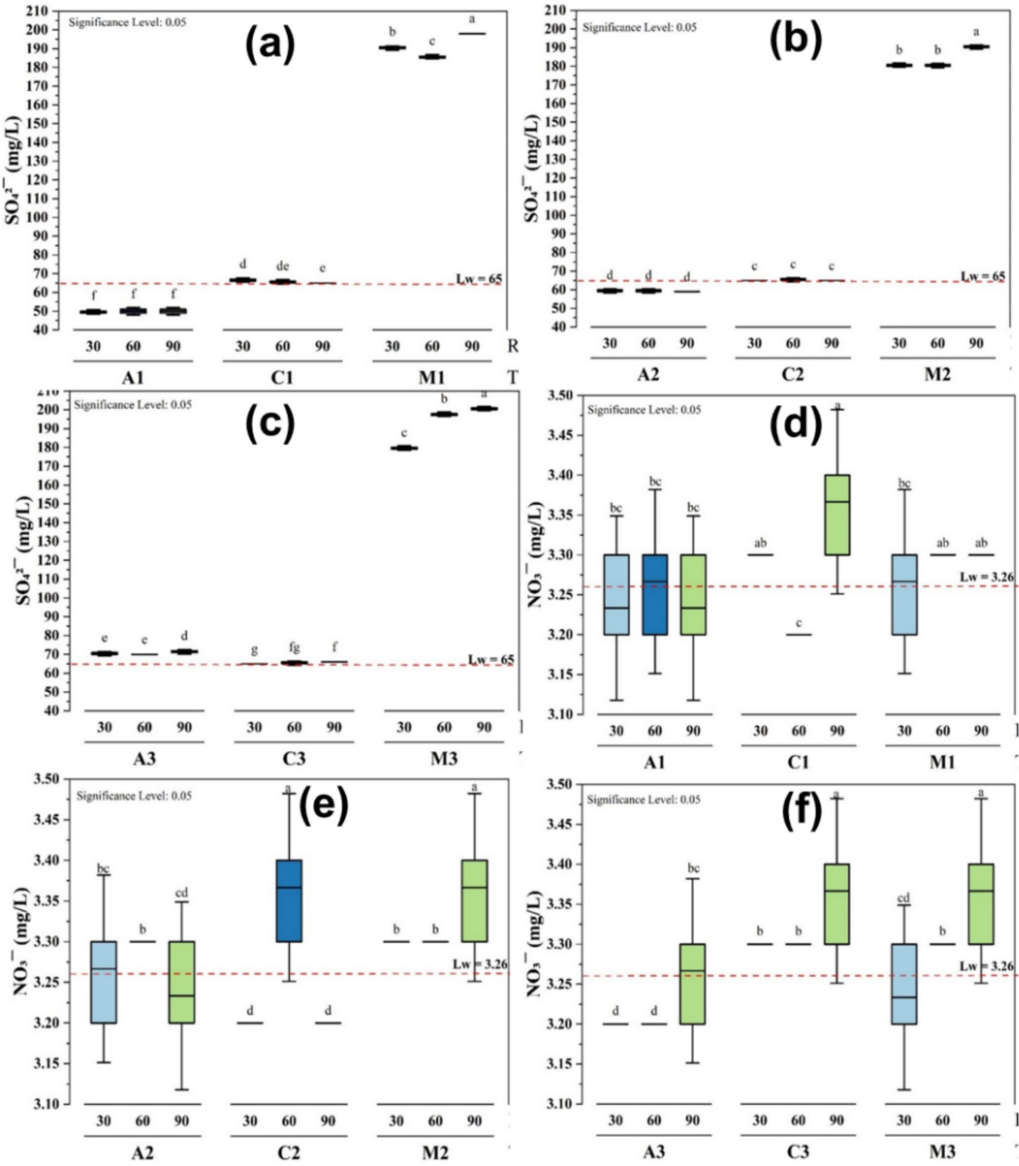
Effects of different treatments (**a**, **d** 25 mL; **b**, **e** 30 mL; and **c**, **f** 35 mL) on the sulfate and nitrate at different coagulant dosage volumes and times of decantation

The effects of different treatments on sulfate and nitrate concentrations were evaluated over 30, 60, and 90 min and different dosage volumes (25 mL, 30 mL, and 35 mL), with the lake water (Lw) serving as the control. The initial sulfate concentration in the water was 65.00 ± 1.00 mg/L, and the nitrate was 3.26 ± 0.06 mg/L, providing a baseline for comparison (Fig. 7). The letters a to d, and a to g show the results of statistical comparisons on nitrate and sulfate, respectively. We only observed a significant difference at p ≤ 0.05 during the treatment with *M. oleifera* on sulfate over time at volumes of 25 mL (Fig. 7a) and 35 mL (Fig. 7c).

After 90 min, the *M. oleifera* treatment caused a substantial increase in sulfate levels, reaching 200.67 ± 0.58 mg/L (Fig. 7c), which was significantly higher than the baseline (p < 0.05), while the lowest sulfate concentration was during the alum treatment with a value of 49.67 ± 0.58 mg/L (Fig. 7a), showing a slight decrease from the baseline value, but this reduction was not statistically significant (*p* > 0.05). All the results were below the 250 mg/L limit recommended by the World Health Organization for drinking water.

Overall, the results indicate that *M. oleifera* significantly increased sulfate concentration over time, with the highest recorded value at 90 min. In contrast, alum consistently reduced sulfate levels, maintaining the lowest values throughout the study. Chitosan exhibited minimal impact, with values fluctuating slightly around the initial concentration. These findings suggest that *M. oleifera* treatment may introduce or release sulfate into the water, whereas alum is more effective in reducing sulfate levels. This can be explained by the chemical composition of *M. oleifera* seed powder made using X-Ray Fluorescence (XRF) technique scan, where the chemical oxide SO_3_ was found to be the main component (Mohseni-Bandpei et al. 2018; Rasheed et al. 2023). The substantial increase in sulfate is attributed to the dissolution of sulfate salts (e.g., K₂SO₄, MgSO₄) inherently present in the seed matrix. Upon addition to water, these highly soluble salts readily dissolve, releasing SO₄^2^⁻ ions. Furthermore, the hydrolysis of alumino-silicate minerals or organic sulfur compounds present in the seed powder during the aqueous extraction and mixing process can contribute additional sulfate ions to the solution (Hashish et al. 2025). This represents a significant trade-off: while *M. oleifera* is effective for turbidity removal, it concurrently leaches dissolved ionic species, increasing the water’s ionic strength as reflected in the concurrent rise in conductivity and TDS**.** The results of the present study are also in agreement with the findings of Herrera et al. (Gutierrez Herrera et al. 2024) who reported that treatment of wastewater with *M. oleifera* increased the concentration of SO_4_^2−^.

The higher concentration in nitrate levels at the volumes of 30 and 35 mL was observed with *M. oleifera* and chitosan at 3.37 ± 0.06 mg/L (Fig. 7e and f), and the lowest at 3.20 ± 0.00 mg/L with alum at the dosage volume of 35 mL (Fig. 7f) and chitosan at dosage volumes of 25 mL and 30 mL (Fig. 7d and e). The values were below the 50 mg/L limit recommended by the World Health Organization for drinking water.

We also observed that the treatments had minimal effects on the nitrate concentration in the water compared to sulfate. Most treatments with chitosan and *M. oleifera* had no significant effect at 30 and 60 min, maintaining the control level of 3.30 ± 0.00 mg/L, while alum caused a slight decrease, but this difference was not significant across time points. By 90 min, most treatments caused only slight increases in nitrate concentrations, with no treatment demonstrating a substantial impact on the nitrate levels compared to the control. This suggests that the treatments had little to no effect on the nitrate concentration in the lake water (Ndabigengesere and Subba Narasiah 1998). In agreement with these findings, a slight elevation in nitrate concentration was also observed in water treated by plant-based coagulants like *M. oleifera *because of their natural content of nitrogen/nitrate (Taiwo et al. 2020). This low level of nitrate during all treatment is good, knowing the bad effects of high nitrate levels in drinking water, like health concerns and water quality problems (Chetty and Prasad 2016; Bishayee et al. 2022).

## Principal component analysis

**Fig. 8:**
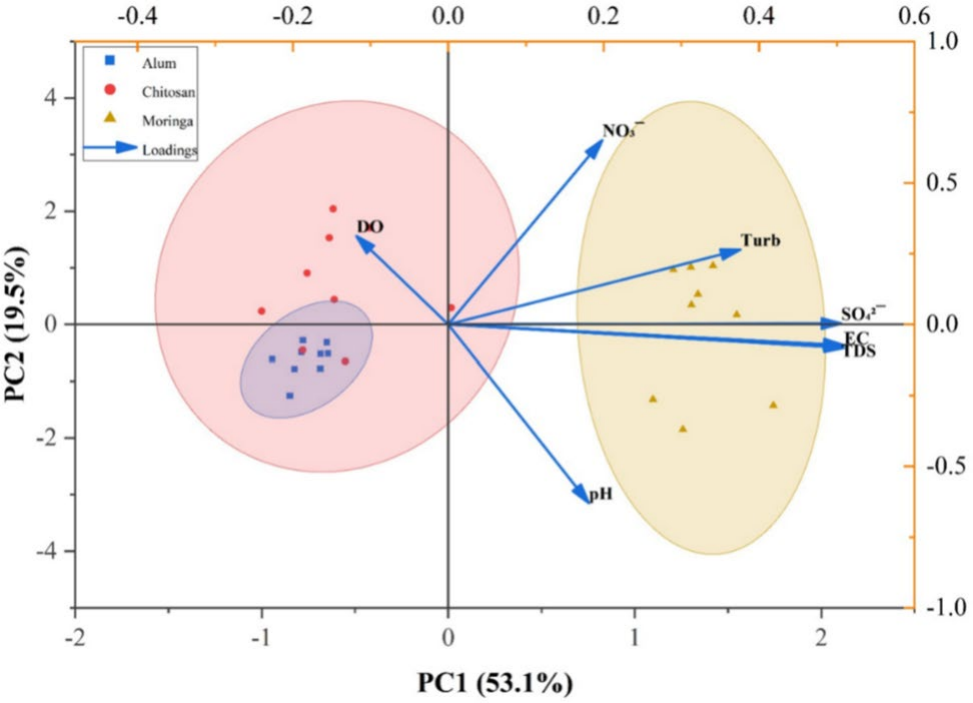
PCA of parameters during different treatments

The principal component analysis (PCA) conducted on the water quality parameters and treatment methods revealed in Fig. 8 that the first principal component (PC1) has the highest value of 4.64149 (53.1%), explaining the largest portion of variance in the dataset. The second principal component (PC2) follows with an eigenvalue of 2.27243 (19.5%), indicating that these two components together account for a significant proportion of the variability observed. This PCA biplot compares the effect of alum, chitosan, and *Moringa oleifera* in water treatment, and it suggests that the primary differences in water quality are influenced by specific parameters and treatment methods.

In terms of variable contributions, PC1 is strongly influenced by sulfate (SO₄^2−^), conductivity (Cond), total dissolved solids (TDS), and pH, which have high positive loadings. These parameters exhibit similar behavior under different treatments, indicating that they are key indicators of treatment efficiency. Treatments also show strong contributions to PC1, suggesting that different coagulants such as alum, chitosan, and *Moringa oleifera* have distinct effects on water quality. This pattern reveals that the primary discriminant among the coagulants is their distinct impact on the ionic composition and acidity of the water, forming a characteristic chemical signature for each treatment type** (**Hassan et al. 2025; Kitheka et al. 2022)**.** On the other hand, PC2 is primarily influenced by retention time (RT) with a value of 2.27243, dissolved oxygen (DO) at 0.70542, and nitrate (NO_₃_^−^) at 0.00564, indicating that these parameters are more sensitive to the duration of treatment rather than the treatment type. This separation indicates that variations in DO and nitrate are governed by time-dependent processes such as settling, re-aeration, or potential biological interactions, which are largely independent of the initial coagulant chemistry (Tambo et al. 2020).

Furthermore, parameters such as pH (1.15726), conductivity (0.12591), and TDS (0.07436) cluster together in PC1, suggesting that they are highly correlated and respond similarly to the treatment processes. This strong correlation validates a fundamental principle in coagulation science: that changes in ionic strength, reflected by conductivity and TDS, are intrinsically linked to shifts in pH due to coagulant hydrolysis and polymer charge interactions (Koul et al. 2022). Turbidity (0.0141) has moderate loadings on both PC1 and PC2, indicating that its variability is influenced by both treatment type and retention time. This dual influence confirms that achieving final clarity is a two-stage process: initial destabilization governed by coagulant-specific chemistry (PC1), followed by floc aggregation and sedimentation which are functions of time (PC2). Meanwhile, dissolved oxygen (DO) and retention time (RT) are closely associated in PC2, implying that oxygen levels in treated water depend significantly on how long the treatment process is applied.

Examining the clustering of treatments, alum and chitosan appear to have similar effects on water quality, as they are positioned near each other in the PCA space, while *Moringa oleifera* demonstrates a distinct behavior. This spatial clustering provides a multivariate confirmation that the synthetic metal salt (alum) and the cationic biopolymer (chitosan) share a dominant coagulation mechanism: charge neutralization exerting a comparable influence on the water’s physicochemical profile (El Foulani et al. 2023; Wang et al. 2017). In contrast, *Moringa oleifera*’s protein-based flocculants operate through a discernibly different pathway, likely involving greater organic bridging or a lesser direct impact on dissolved ionic species (Vigneshwaran et al. 2020). This differentiation highlights that the effectiveness of these coagulants varies in terms of altering water quality parameters. The results also suggest that water quality improvements are primarily driven by the type of treatment used, as reflected in PC1, while retention time plays a secondary role in influencing parameters like dissolved oxygen and nitrate levels, which are captured in PC2.

Overall, the PCA results show that treatment type and retention time are the main factors influencing variations in water quality in the dataset. Indicators of treatment effectiveness include pH, conductivity, and sulfate, whereas dissolved oxygen and nitrate are more reliant on treatment duration. The analysis fundamentally distinguishes between coagulant-driven chemical effects (PC1) and time-dependent physical processes (PC2). It further reveals an unexpected mechanistic similarity between alum and chitosan in this system, contrasting with the distinct behavior of the plant-based *Moringa oleifera*. These results offer insightful information on the effectiveness of various treatment techniques and how they affect the quality of the water.

While alum offers performance reliability for engineered systems, the field-scale practicality of *Moringa oleifera* is high in resource-limited contexts due to local availability, low sludge volume, and minimal pH alteration, provided sulfate increase is managed. Chitosan occupies a middle ground, suitable for systems that can handle its pH-dependent preparation. This study provides the essential efficacy data to inform these larger-scale practical decisions.

## Conclusion

This study evaluated the use of *Moringa oleifera* seeds, chitosan, and alum for the treatment of lake water. All the treatments reduced the turbidity by a percentage greater than 95% at a certain dosage and after a specific settling time. In addition, *M. oleifera* seed-derived coagulants did not significantly change the pH of the treated water, unlike chitosan and alum, which showed a reduction in pH. Moreover, the *Moringa oleifera* and chitosan treatments exhibited the most consistent improvement in DO, while the alum treatment showed a moderate but steady increase in DO. Besides, it was noted that *Moringa oleifera* exhibited the most significant impact on increasing conductivity, followed by chitosan, whereas alum had the least impact on conductivity. *M. oleifera* also significantly increased sulfate concentration over time, but chitosan exhibited minimal impact, and alum was more effective in reducing sulfate levels. On the other hand, all the treatments had minimal effects on the nitrate concentration in the water. This study is the first one where *Moringa oleifera* seeds and chitosan are used to explore their potential in treating lake water in Johannesburg, providing a comparative evaluation of the efficacy of the different adsorbents. *Moringa oleifera*, being a cheap and readily available natural resource, is a sustainable option for use as an adsorbent in water treatment applications. Future work should isolate Moringa’s active proteins to mitigate salt leaching and develop modified chitosan for enhanced adsorption. Pilot-scale trials are needed to evaluate real-world performance, sludge management, and cost, advancing these sustainable options for application.

## Supplementary Information

Below is the link to the electronic supplementary material.

ESM1 (XLSX 18.1 KB)

## Data Availability

The data supporting this article have been included as part of the Supplementary Information.
